# A New Theory in Regard to the Function of the Duodenum

**Published:** 1887-12

**Authors:** 


A New Theory in regard to the Functions of the Duodenum.—Treves has observed that the third portion of the duodenum is firmly attached to the four lumbar vertebræ by a ligament called the musculus suspensorius duodenalis. This fact is observed pretty constantly in animals and in man, also that the duodenum forms a curve something like a siphon trap. The fixed portion always being stationary, allows the free portion to assume varying degrees of curvature. The duodenum being always more or less filled with fluid from liver and pancreas, that this curving of the duodenum performs the function of a siphon trap, and absorbs all the fetid gases that form in the bowels, that might have a tendency to regurgitate upwards.—*Weekly Medical Review.*

